# DPD Modelling of the Self- and Co-Assembly of Polymers and Polyelectrolytes in Aqueous Media: Impact on Polymer Science

**DOI:** 10.3390/polym14030404

**Published:** 2022-01-20

**Authors:** Karel Procházka, Zuzana Limpouchová, Miroslav Štěpánek, Karel Šindelka, Martin Lísal

**Affiliations:** 1 Department of Physical and Macromolecular Chemistry, Faculty of Science, Charles University, Hlavova 8, 128 43 Prague, Czech Republic; zl@natur.cuni.cz (Z.L.); miroslav.stepanek@natur.cuni.cz (M.Š.); 2 Department of Molecular and Mesoscopic Modelling, Institute of Chemical Process Fundamentals, Czech Academy of Sciences, Rozvojová 135, 165 02 Prague, Czech Republic; sindelka@icpf.cas.cz (K.Š.); lisal@icpf.cas.cz (M.L.); 3 Department of Physics, Faculty of Science, Jan Evangelista Purkyně University in Ústí nad Labem, Pasteurova 3632, 400 96 Ústí n. Labem, Czech Republic

**Keywords:** polymer, polyelectrolyte, assembly, dissipative particle dynamics

## Abstract

This review article is addressed to a broad community of polymer scientists. We outline and analyse the fundamentals of the dissipative particle dynamics (DPD) simulation method from the point of view of polymer physics and review the articles on polymer systems published in approximately the last two decades, focusing on their impact on macromolecular science. Special attention is devoted to polymer and polyelectrolyte self- and co-assembly and self-organisation and to the problems connected with the implementation of explicit electrostatics in DPD numerical machinery. Critical analysis of the results of a number of successful DPD studies of complex polymer systems published recently documents the importance and suitability of this coarse-grained method for studying polymer systems.

## 1. Introduction

The self-assembly of amphiphilic block copolymers and the co-assembly of double hydrophilic block polyelectrolytes are important phenomena that have been studied by experimentalists, theoreticians and computer scientists for several decades [1,2,3,4,5,6,7,8,9,10,11,12,13,14]. Self- and co-assembled nanoparticles find numerous applications in biomedical fields, e.g., as carriers in the targeted delivery of drugs or genes in living organisms, as important agents in radiotherapy and photodynamic therapy and as promising biomarkers and biosensors [14,15,16,17,18,19,20,21,22,23,24,25]. Simultaneously, their applications are increasing exponentially in other fields comprising various technologies and environmental applications. They find use in catalytic systems [26,27,28,29,30], in the preparation of inorganic nanoparticles, where they serve as templates for their self-assembly [31], in the fabrication of integrated circuits, photoconductive devices and more [24,32,33,34]. The number of relevant experimental studies aiming at either the basic principles and trends of polymer self-assembly or at their important applications is so vast that it is impossible to cite all important studies, and we mention above only a small fraction of the papers published.

In spite of the fact that high-molar-mass copolymers containing a long and strongly hydrophobic block, such as a polystyrene, poly(methyl methacrylate), poly(1,4-butadiene) or polyisoprene block, and a comparably long water-soluble poly(acrylic or methacrylic acid), poly(2- or 4-vinylpyridine), poly(ethylene oxide) or polyoxazoline block are insoluble in aqueous media, experimentalists soon revealed that the stable aqueous dispersions of their self-assembled nanoparticles can be prepared indirectly [4,5,35,36,37,38,39,40] if the copolymer is first dissolved in a common solvent for both blocks, i.e., in an aqueous mixture rich in a suitable organic solvent. The second step assumes either (i) dialysis against aqueous buffers or (ii) selective evaporation of the organic solvent. A third method (iii) proposed a few years later (called ‘quenching’) consists of the fast injection of the copolymer dissolved in an organic solvent-rich mixture into an excess of the aqueous phase (water or an aqueous buffer) under vigorous stirring [5,36,37]. We purposely discuss the methods of preparation of aqueous micellar dispersions to remind readers that a high percentage of seminal experimental studies on copolymer micelles with cores formed by hydrophobic high-molar-mass and high-*T*_G_ (glass transition temperature) blocks, such as polystyrene or poly(methyl methacrylate), concern the kinetically arrested systems, the properties of which do not correspond to reversible associates in aqueous media.

The decisive force driving the formation of polymeric micelles derives from the appreciable decrease in enthalpy upon the minimisation of unfavourable interactions of insoluble blocks with solvent molecules. However, this contribution does not control the association number, shape or inner structure of the associates [41,42,43,44,45,46,47]. The ‘stop-growth’ factor controlling the structural characteristics is the entropy aiming at the maximum disorder, i.e., at associated structures enabling the maximum numbers of chain conformations in cores and in shells. Because the enthalpy drives the process but the intricate entropy-to-enthalpy interplay (dominated by entropy) controls the structural characteristics, the self-assembly should be classified as the enthalpy-driven and entropy-controlled process.

The double-hydrophilic block copolymers containing a long polyelectrolyte (PE) and a long neutral water-soluble block are the other class of application-promising materials exploitable in medicine and in various nanotechnology fields [48,49,50,51,52,53]. In aqueous solutions, they assemble into nanoparticles that contain one or a few insoluble inter-polyelectrolyte complex (IPEC) domains stabilised by hydrated domains formed by the water-soluble blocks [54,55,56,57,58,59,60,61,62]. The association proceeds spontaneously upon mixing the solutions of oppositely charged double-hydrophilic copolymers or upon mixing the solutions of double-hydrophilic copolymers with oppositely charged homopolymers.

Even though the electrostatic interactions are the prerequisite of the co-assembly, the driving forces of the process do not derive from electrostatics. First, the Coulomb force between two charges, *q*_1_ and *q*_2_, kept at a certain distance *r* from each other, is proportional to *q*_1_*q*_2_/*r*^2^ and depends on the electric permittivity of the medium *ε*, but it does not depend on whether the charged species are counterions or pendant groups on the PE chain. Second, the electric charges on PE chains are fairly well compensated by the cloud of counterions, which, depending on the density of PE charges, either move in the immediate vicinity of chains or partly condense on PE chains [63] (the Manning condensation). The cooperative effect of multiple interactions between oppositely charged PE chains and a relatively low local *ε* in proximity to nonpolar chains promote the association process, but the change in the electrostatic interaction energy is small and a considerable increase in entropy [64] upon the formation of IPEC domains and the release of mobile counterions into the bulk solvent drives the association. Contrary to intuitive expectation, the structural characteristics of associates are neither controlled by electrostatics nor by the entropy increase. The association number, size, shape and their inner structure depend mainly on the hydrophobicity/hydrophilicity of PE blocks, on their compatibility with neutral water-soluble blocks and further on their lengths, flexibility and interactions with small ions [65,66,67,68,69].

In the last two decades, various composite nanostructured materials and multicompartment micelles have been prepared by the simultaneous or sequential application of several self- and/or co-assembling steps discussed above, their structure and properties have been studied experimentally, theoretically and by computer simulations, and the most important trends of their behaviour have been described in the literature [3,5,7,8,12,14,70,71,72,73,74,75,76,77,78,79,80,81,82,83].

In this review article, we describe the general principles and important results of the coarse-grained computer modelling of the self- and co-assembly of block copolymers and PEs in aqueous media, focusing on the recent progress achieved with this method and on its impact on polymer science. We discuss the most important papers on the polymer and PE self-assembly published in recent decades both with and without the explicit electrostatics. Our aim is to show the parallels between the assumptions used in DPD and those used in polymer physics. In contrast to an excellent methodology-orientated recent review by Neimark [84], we focus on DPD results elucidating intrigue and problems of polymer chemistry that are not yet fully understood. The main goal is to inform a broad community of polymer scientists on the advantages and benefits of coarse-grained computer modelling.

## 2. Coarse-Grained Computer Modelling of Polymer Chains

Since the early application of Monte Carlo simulations by Ulam and his colleagues in the Manhattan Project more than 70 years ago, computer modelling and computer simulations in particular have become important tools of physical, chemical, biological and material research [85,86,87,88,89]. They enable checking of the correctness of theoretical hypotheses and predictions and provide data that are either inaccessible or barely accessible by experiments. Furthermore, in combined experimental and theoretical research, they offer a relatively fast and cheap substitute for a substantial part of the tedious and expensive experimental work [90,91,92,93,94]. Thanks to enormous progress in computer technology, atomistic simulations (even quantum simulations) of relatively large systems, such as enzymes and catalytic or biocatalytic centres, have become feasible in recent decades [95,96,97,98,99].

The huge size and complexity of self-assembling polymer systems exceed the limits of up-to-date atomistic simulations. A reasonable computer study of systems of interest assumes simulations of hundreds of relatively long chains immersed in a pool of thousands of solvent molecules. Moreover, the time and length scales of the dynamic processes controlling the behaviour of self-assembling systems, which cannot be neglected in atomistic simulations, span more than ten orders of magnitude. Successful research on associating polymer systems thus calls for the use of gross coarse-grained simulation methods [100]. In this review, we focus on one particular coarse-grained variant of molecular dynamics (MD) called dissipative particle dynamics (DPD). The principle of DPD and all necessary details of the computational machinery, including the parameter setting, are outlined in seminal papers by Groot, Warren, Español and others [101,102,103,104] and in several reviews [84,105,106,107], and in the papers cited therein. Another review on the coarse-grained modelling of soft-matter systems, including DPD simulations, was presented by Holm and his collaborators; see, e.g., Refs. [108,109,110,111]. Practically, DPD simulations can be carried out within popular particle-based simulation packages such as LAMMPS [112] or with coarse-grained simulation packages such as DL_MESO [113], ESPResSo [114] or HOOMD-blue [115]. Here, we focus on the simulation results contributing to the deepening of the knowledge of the polymer self-organisation and self-assembly and on results indicating novel promising applications. Nevertheless, in the following paragraphs, we briefly outline and discuss the basic technical features of DPD simulations necessary for understanding the text.

In contrast to atomistic simulations and to fine coarse-grained methods, which study the assemblies of molecules, DPD focuses on the behaviour of larger lumps of studied systems, i.e., on clusters of several solvent molecules, polymer segments, solvated ions, etc., capitalising on the fact that larger parts of studied systems (called DPD beads) behave as soft and mutually (more or less) interpenetrating objects that facilitate the numerical treatment. DPD addresses the global behaviour on relatively large (mesoscopic) length scales and on slow time scales, which means that a number of fast motions and short-range interactions have been sacrificed for the sake of fast and smooth computations. However, their effect cannot be neglected and is accounted for by the dissipative and random forces linked with the fluctuation–dissipation theorem (FDT), which reflects the interplay of the ensemble-average friction (which slows down the motion of DPD beads) versus the overall thermal agitation (which accelerates the beads). The balance of dissipative and random forces then serves as thermostat in DPD systems [116]. Analogously to other MD methods, the DPD computational machinery consists of a numerical solution of the Newtonian equations of motion for a system of DPD beads (*i*, *j*, *k, …*) that mutually interact by conservative forces *F*_c_(*r_ij_*) derived from the soft repulsive distance-dependent pair interaction potential *U*_c_(*r_ij_*). The DPD equations of motion are typically integrated by modified Verlet algorithms [117]. They allow the employment of values of the integration timestep Δt of around 0.5 in reduced units for DPD fluids, which are approximately an order of magnitude larger than those for fluids modelled by hard-core potentials such as the Lennard-Jones potential. The values of Δt need to be slightly reduced, typically to 0.01, when polymer models involve additional rigidities such as stiff bonds or stiff bendings [118]. Larger values of Δt can be adopted when advanced integration algorithms, such as Shardlow splitting algorithms [119], are used. However, the advanced integration algorithms are more computationally intensive with respect to the modified Verlet algorithms. The stronghold and practical computational advantage of DPD lie in the fact that the soft interparticle forces neither change abruptly nor diverge at short distances, which permits long integration steps as compared to atomistic simulations. This fact appreciably accelerates the simulations and, together with the gross coarse-graining, allows for the study of mesoscale systems. The disadvantage of the coarse-graining and high simulation speed is a loss of detail and a slightly foggy picture of the behaviour. Nevertheless, if the model reproduces the essential features of the system well and the forces are set correctly, DPD enables physically sound studies of complex systems, which cannot be reasonably performed by any other simulation method. Another stronghold of DPD consists in the fact that the short-range friction effect is treated explicitly at the molecular level. In short, DPD is a powerful and versatile method for studying large systems but its use covers non-negligible risks that have to be avoided. Before attempting a DPD study, it is necessary to identify the properties that can be studied safely by the coarse-grained method. Bearing the above message in mind, one has to analyse the simulation results carefully with all due prudence and withstand the temptation to interpret unsafe, tiny details.

Polymers are modelled in DPD as flexible, freely jointed chains or semiflexible chains, e.g., worm-like chains [120], formed by DPD beads interconnected by ‘springs’ emulating the covalent bonds (bead-spring model). The bonds connecting two neighbouring beads in polymer chains are described by a combination of the stretching potential, e.g., the harmonic (Hookean) potential [121,122], FENE (finitely extendible non-linear elastic) [123] or Morse potential [124] and a soft repulsion potential. In studies of semiflexible chains, bond angles (harmonic, harmonic cosine or cosine) [125,126,127,128] and/or dihedral bond potentials are also used. The choice used most often is the combination of the harmonic bond stretching potential, soft repulsion potential and the bond angle potential. An interesting alternative approach for modelling the restricted chain flexibility was used recently by the Neimark group and will be discussed later in more detail [121].

The optimum setting of interaction and bond parameters is the key condition for any successful DPD simulation; therefore, it was investigated in several papers [123,125,129,130,131,132,133] At first glance, it is surprising that simulations using fairly different values of bond and interaction parameters provide almost identical descriptions of the behaviour of polymer systems. We will discuss and explain this apparent ‘paradox’ in the following sections. First, we focus on the construction of covalent bonds. Some authors use harmonic potentials with a low spring constant and zero equilibrium distance, which they combine with a relatively weak repulsion (comparable with other forces), i.e., the springs are fairly stretched [134]. Other authors prefer the spring potential with nonzero equilibrium distance and a high spring constant between the mutually bonded beads [125,129]. As the average bond stretching is similar in both cases and the local flexibility and conformational behaviour of the chain are predominantly controlled by other parameters (e.g., by the bond angle potential), the approaches differing in the modelling of the bond stretching provide highly comparable simulation results. Strictly speaking, the former choice generates a chain that is slightly more deformable than that based on the latter setting, but the difference in the conformational behaviour is negligible at the coarse-grained level.

This review addresses a broad community of polymer scientists, the overwhelming majority of whom are not experts in coarse-grained computer simulations. Therefore, we feel obliged to explain three important facts, two arising from the definition and treatment of non-electrostatic DPD forces and the third from the stability of numeric computations.

First, the original DPD method developed for nonpolar systems without electrostatics and without specific interactions employs only two types of conservative forces: repulsive forces acting between chemically non-bonded beads and forces emulating chemical bonds in polymer chains, i.e., attractive forces between non-bonding beads, are missing. The repulsive forces drop to zero at a cut-off distance, *r*_C_, which defines the size of the soft bead. Such a strange ‘force field’ could be confusing at first glance for a number of polymer scientists, but this concept is reminiscent of the Flory–Huggins (FH) approach, in which the *χ* parameter compares the space-limited cross-interaction between components *i* and *j* with the average of their like interactions *ii* and *jj*. Analogously to the FH theory, a weaker repulsion between some components than that acting between other components emulates the attractive DPD force.

Second, as all forces (in common non-electrolyte systems) are repulsive, the beads representing the solvent exercise pressure *p* on the walls of the simulation box. This fact enables the calibration of the repulsion forces of the studied system on the basis of the dependence of the solvent compressibility, *p*/(*ρk*_*B*_*T*), on density, *ρ* (*k*_*B*_ is the Boltzmann constant and *T* is the system temperature). As most DPD simulations address aqueous solutions, the experimental dependence of the the comressibility on density of water is commonly used for the calibration.

**Figure 1:**
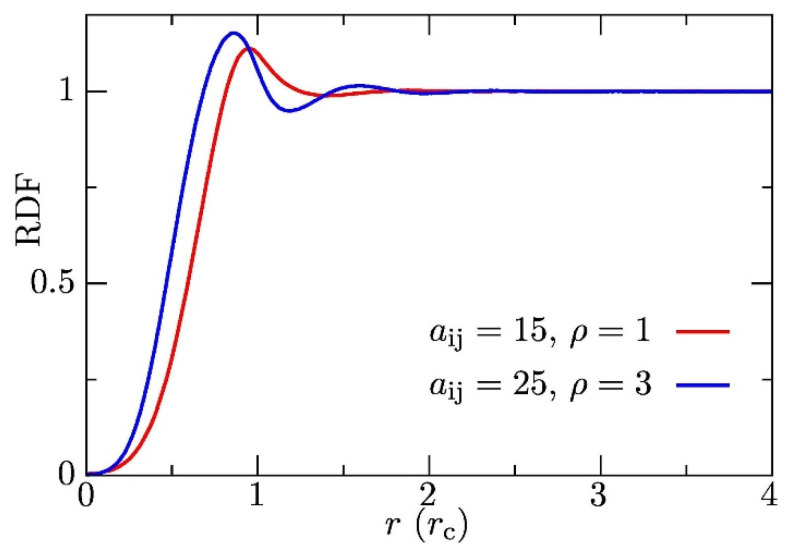
Radial distribution function for *ρ* = 3 (blue solid line) and *ρ* = 1 (red solid line).

Third, the overwhelming majority of published DPD studies have been performed at a relatively high density of beads, *ρ* = 3 (in reduced DPD units, i.e., 3 beads in volume (*r*_C_)^3^), which could be highly confusing for most experimentalists. The reason for using the elevated density is simple. The stability of the numerical solution is much better at higher densities than *ρ* = 1. However, such an explanation would not satisfy the experimentalists. Fortunately, the statement can be supported by clear physical arguments. (i) As the calibration of repulsion forces is based on the density-dependent experimental compressibility, the results of DPD simulations practically do not depend on the density (within the limits of exploited simplifications). The higher the density, the stronger the repulsions between the beads. Because this rule applies for all components of the system (the solvent as well as the solutes), the strengths of solvent–solvent, solvent–solute and solute–solute interactions vary analogously, which means that the global result of their interplay remains almost the same. (ii) Real liquids, and water in particular, are barely compressible as a result of the excluded volume of molecules. The DPD beads are soft and can partly interpenetrate each other, which means that, at low densities, they do not feel the excluded volume effect strongly enough. Even though the whole volume of the simulation box is filled in by soft spherical beads at densities of around 1, the beads only touch each other and almost do not overlap. As the inter-bead interaction potential drops to zero at the cut-off distance defining the size of the DPD bead, the interaction energy of the whole system is very weak. At the elevated density *ρ* = 3, the bead centres are relatively close to each other and important repulsive forces between individual beads partially emulate the excluded volume effect, i.e., the system of DPD beads behaves as a compressible liquid. This is documented by the fact that the simulated radial distribution function for *ρ* = 3 shows a sign of damping oscillations and is more reminiscent of that of real liquids than the RDF for *ρ* = 1 (see Figure 1). Furthermore, Groot and Warren have shown, in their seminal papers [101,102,103,104], that, starting at *ρ* = 2, the increment in the compressibility factor with density remains constant, which indicates a self-consistent compressibility behaviour.

The questions as to whether the DPD method is scale-free and applicable in a wide range of length scales or if and how the parameters scale with the size of DPD beads (i.e., with the number of solvent molecules forming one bead) have been the subject of several studies [101,135,136]. The results of these studies, supported by a number of specific application-orientated papers, indicate that the DPD simulations of static equilibrium properties of polymer systems (including the studies of the polymer self-assembly) based on the mass of the bead, *m*, cut-off radius, *r*_C_, energy in *k*_*B*_*T* and the time unit defined as *τ* = *r*_C_[*m*/(*k*_*B*_*T*)]^1/2^ correctly reproduce the experimental behaviour in the whole mesoscopic range, provided that the relative differences between interactions of individual components have been set appropriately. However, the simulations of dynamic properties, which are not a topic of this review, require the appropriate setting of parameters, particularly the recalculation of the time unit at a given coarse-grained level [102,137,138,139].

## 3. DPD from the Viewpoint of Polymer Physics: Mapping the DPD Model onto a Realistic Model of Polymer Systems

**Figure 2:**
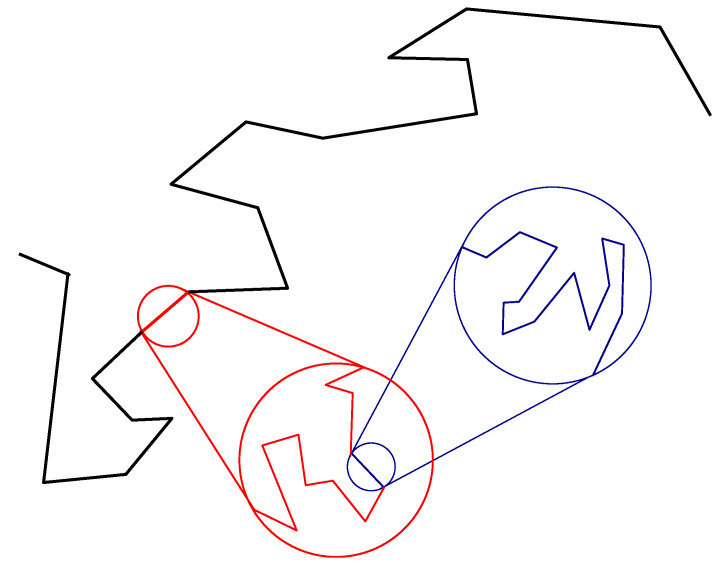
The schematic depicts fractal properties of polymer chains and simultaneously outlines the principle of coarse-graining modelling of polymers, illustrating thus the linkage between the simplifying model assumptions and the real behaviour.

DPD simulations are a very suitable tool for studying polymer chains, not only because the polymer chains are long and their study requires the coarse-grained approach, but mainly because the simplifying assumptions used in DPD respect the real behaviour of polymer chains and are similar to those currently used in polymer physics. The classical polymer theories treat polymer chains as a string of short linear segments that are connected to each other, but their unrestricted or only partially restricted mutual orientations allow for astronomically high numbers of conformations. The segments are characterised by the length and by the excluded volume, which depends on interactions with other segments and with the solvent. The overwhelming majority of theories of polymer solutions [64,140,141] assume that one segment comprises several monomeric units, because the fixed valence bond angles and barriers of hindered rotation restrict the set of mutual orientations of two neighbouring monomeric units and only a short part of the chain (5–7 units, depending on the complexity of their chemical structure) provides the flexibility required by the models. Flexible polymer chains generally form random coils in dilute solutions (if their behaviour is not affected by strong electrostatic or specific interactions). In *θ* solvents, when the excluded volume effect of segments is compensated by relatively mild, attractive segment–segment interactions (analogy of the Boyle temperature for gasses), the density of segments in the radial distance from the gravity centre follows the Gaussian curve and the coils behave as entropic springs, i.e., the energy needed for their expansion or compression is proportional to the increase or decrease in the mean-square end-to-end distance, respectively. The average density of segments in the coil is low in dilute solutions, and the volume occupied by segments reaches only a few percent of the coil volume depending on the chain length and solvent quality. In good solvents, the chains expand (swell and the segment density decreases), and, in poor solvents/non-solvents, they shrink and finally collapse. It should be stressed that the random coils formed by high-molar-mass polymers do not obey the scaling laws of trivial 3D (constant density) objects. They are nontrivial fractal structures containing the self-similar motifs replicating at several length scales (Figure 2), and a number of their unique features arise from the very high flexibility of their parts at various length scales.

When the polymer concentration increases and exceeds the concentration at which the polymer coils fill in the whole volume and touch each other (concentration of the first overlap, c*), the coils do not shrink, but they start to interpenetrate, which is accompanied by sharp changes in the solution properties (e.g., by a huge increase in viscosity).

This shows that the whole polymer coils and their shorter parts, down to the level of Kuhn segments, behave as soft, interpenetrating objects and meet the basic assumptions of the DPD method very well. These facts were soon realised by the pioneers of DPD, Groot and Warren, who applied the method on nonpolar polymer systems and mapped DPD results onto the FH systems, which enabled them to derive a simple formula for the recalculation of the Flory interaction *χ* parameters into DPD parameters of inter-bead conservative forces.

(1) $$\chi_{ij}=2\;\alpha (r)\;\;\rho \;r_{c}^{3}\;\left(a_{ij}-\frac{a_{ii}+a_{jj}}{2}\right)\;\frac{r_{c}}{k_{B}T}$$

where ρ is the total particle density, *r_c_* is the cut-off distance, and α(r) is a parameter dependent on ρ. For *ρ* = 3, Equation (1) acquires the simple form

(2) $$\frac{a_{ij}r_{c}}{k_{B}T}=\frac{a_{ii}r_{c}}{k_{B}T}+3.27\chi_{ij}$$

where *a_ii_* = 75/*ρ*.

By this choice, the authors implicitly set the DPD polymer bead identical to the FH segment (roughly corresponding to the Kuhn segment), even though they have not stressed it explicitly (and have not redefined its mass and volume with respect to the solvent). The success of DPD studies of polymer systems proves that this slight inconsistency does not affect the results and confirms the flexibility of DPD applications [142,143,144,145,146,147,148,149,150,151,152,153,154,155]. This inconsistency arises from the FH lattice theory itself, which postulates that the lattice sites are either occupied by polymer segments or by the solvent, assuming that the sizes of the polymer and solvent segments are comparable (viz. the calculation of molar and volume fractions). As the FH approach provides a sound description of polymer solutions and overcomes the problem of the considerable size asymmetry of components, we believe that this legitimises the FH-based parameter setting with all consequences. For the readers’ convenience, we recall that the *χ* parameter for a good (athermal) solvent, *χ* = 0, translates into *a_ij_* = 25; *a_ij_* = 26.635 describes the *θ* solvent with *χ* = 0.5, and *a_ij_* = 40 describes a poor solvent (nonsolvent) for density *ρ* = 3 in a system where the solvent bead comprises three molecules. Note that the interaction parameters used in computer simulations (not only in DPD) are generally higher than those estimated experimentally for a given polymer because the simulated chains are appreciably shorter than the real ones. Higher values in simulations are theoretically justified by the condition for the phase separation of two incompatible polymers, *Nχ* = 10. This equation shows that the effective chain repulsion depends on the product of the chain length, *N*, and the interaction parameter for one bead, *χ*. The shorter the chains, the higher the *χ* value needed to model the same effect of polymer compatibility/incompatibility.

At present, the parameter setting has been significantly ramified and there are methods regarding the chemical nature of the components [129] and DPD variants enabling the simulation for mixtures of species, the beads of which differ in mass and size [156,157,158,159].

## 4. Electrostatics in DPD

Electrostatics were implemented in DPD by Groot and others [160,161,162]. The common treatment of electrostatic interactions in polymer physics assumes that the charged PE bead bears the elementary charge *e* and most DPD simulations of polyelectrolytes obey this rule. Nevertheless, the treatment of electrostatic interactions in DPD is not straightforward. In contrast to simulations that use the Lennard-Jones and the Coulomb potentials, both strongly diverging at short distances, the application of the Coulomb potential is prohibited in DPD because its use in combination with soft and non-diverging repulsion potentials would cause catastrophic non-physical consequences (the collapse of the oppositely charged beads on top of each other). Therefore, various tricks, e.g., a cut-off of the Coulomb potential at short distances, addition of an impermeable core into the centre of the bead or addition of a narrow impermeable cylinder emulating the bond [148,149,152,153,154,163,164] and others, have been used.

Besides the solution of problems due to the divergence of electrostatic interactions, the addition of impermeable domains also precludes the prohibited intersection of self-avoiding chains. Note that neither the soft beads nor the springs representing the bonds prevent the passage of one polymer chain though the other, and a simple DPD variant does not emulate the entanglements that non-negligibly influence the rheology and viscoelastic behaviour of real high-molar-mass polymers. Another approach, which significantly suppresses the intersection of self-avoiding chains, consists of the addition of the spring–spring repulsion to the bead–bead repulsion [153].

Thus far, a slight delocalisation of charges described by a common Slater-type exponential charge distribution [161]

(3) $$f\left(r\right)=\frac{q\;e}{\pi \;\lambda^{3}}\exp\left(-\frac{2r}{\lambda }\right)$$

where q is the charge fraction, *e* is the electron charge and λ is the charge decay length, has been used by most authors studying PEs by DPD. This treatment is inspired by the fact that (i) the beads do not represent single atoms but larger objects and (ii) the potential describing the interaction of delocalised (smeared) charges does not diverge at short distances. In this case, the interaction between charged particles i and j is given as

(4) $$u_{ij}^{el}=\frac{q_{i}q_{j}}{r_{ij}}\lambda_{B}k_{B}T\left[1-\exp\left(-\frac{2r_{ij}}{\lambda }\right)\left(1+\frac{11r_{ij}}{8\lambda }+\frac{3r_{ij}^{2}}{4\lambda^{2}}+\frac{r_{ij}^{3}}{6\lambda^{3}}\right)\right]$$

where $\lambda_{B}=e^{2}/\left(4\pi \varepsilon_{0}\varepsilon_{r}k_{B}T\right)$ is the Bjerrum length ($\varepsilon_{0}$ is the dielectric constant of a vacuum and $\varepsilon_{r}$ is the relative permittivity of the reference medium), and *q_i_* and *q_j_* are their electric charges. Equation (4) can be reasonably approximated by

(5) $$u_{ij}^{el}=\frac{q_{i}q_{j}}{r_{ij}}\lambda_{B}k_{B}T\left[1-\left(1+\beta r_{ij}\right)\exp\left(-2\beta r_{ij}\right)\right]$$

where β=5/(8λ). Equation (5) provides good accuracy at all relevant distances and is slightly less computationally expensive when compared with Equation (4) and was therefore used in a number of simulations.

Nevertheless, other types of charge smearing (e.g., linearly or exponentially decreasing, Gaussian, etc.) have also been tested [160,162], and the published papers further differ in the extent of delocalisation [134,161] and in the mathematical treatment of electrostatic interactions [161,162]. Concerning the extent of charge delocalisation in Equation (3), a low smearing constant, *λ* = 0.2 (spatially restricted delocalisation), ensures that the whole charge (>99%) remains inside one bead. We believe that this delocalisation reflects the entity of PE segments and small ions and realistically models the interactions and correctly emulates the behaviour of real PE systems, which are controlled by the electrostatic vs. entropic interplay and in which the entropy of small ions plays a crucial role. Nevertheless, it is fair to say that larger spatial charge delocalisation (*λ* = 0.67) [161] also emulates the behaviour of the PE solution well, which suggests that the degrees of delocalisation λ∈(0.2–0.7) do not dramatically change the DPD results.

**Figure 3:**
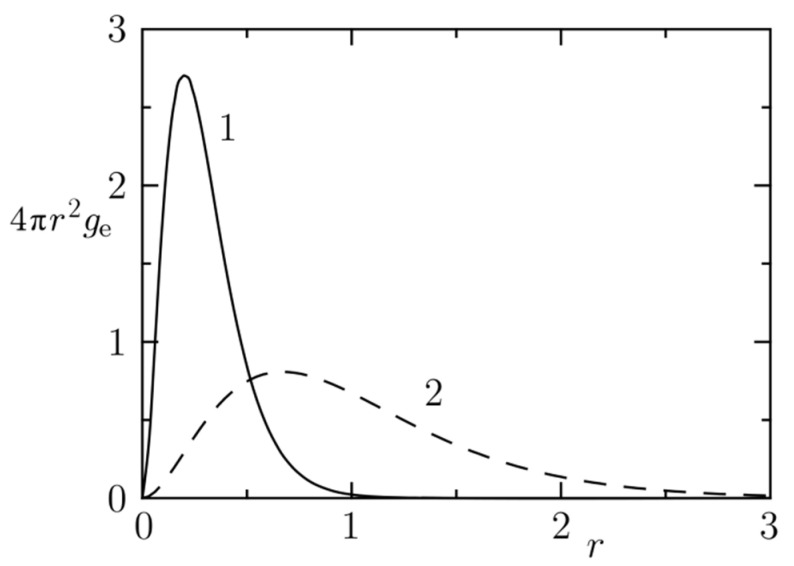
Spatial density *g*(*r*) of the exponentially smeared charge for two decay constants *λ* = 0.20 (1) and *λ* = 0.67 (2). Reproduced from *Phys. Chem. Chem. Phys.* **2016**, *18*, 16127 https://pubs.rsc.org/en/content/articlelanding/2016/cp/c6cp00341a, accessed on 15 December 2021 (supporting information) with permission from the PCCP Owner Societies.

**Figure 4:**
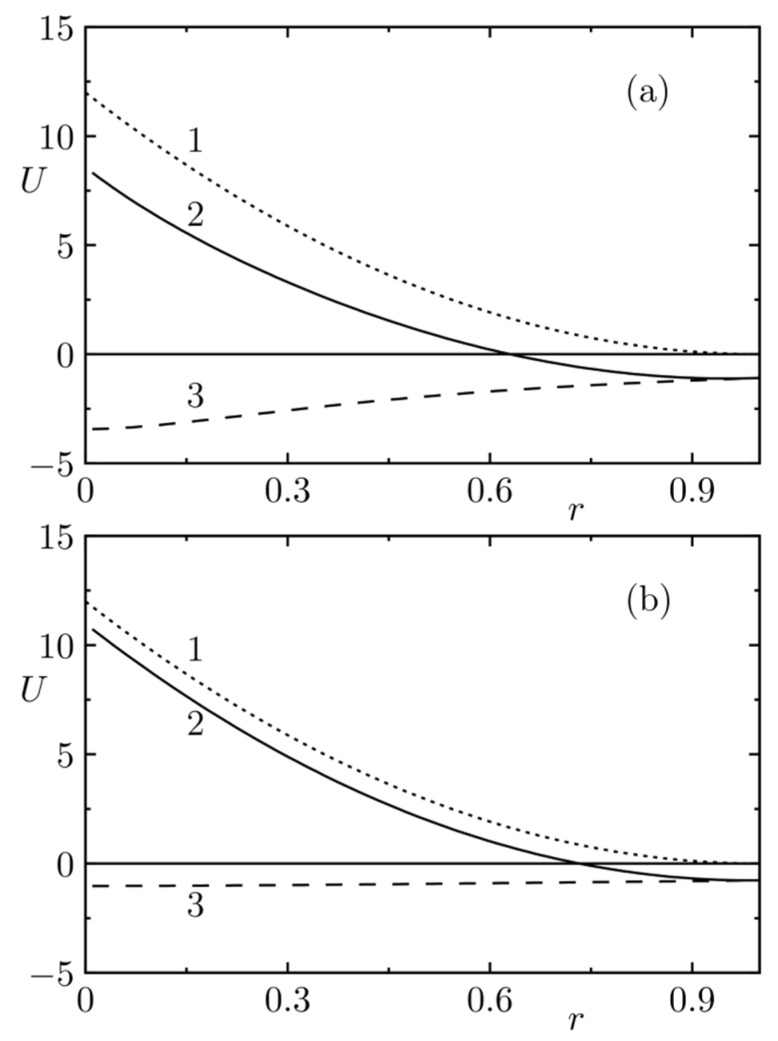
The interaction between oppositely charged beads of the same type, i.e., the sum of the DPD repulsive potential with *a_ij_* = 25 and the smeared electrostatic potential of two oppositely charged clouds (total charges of clouds +*e* and −*e*) for *λ* = 0.2 (**a**) and *λ* = 0.67 (**b**). The full lines (2) represent the sum of the electrostatic (3) and repulsive potentials (1). Reproduced from *Phys. Chem. Chem. Phys.* **2016**, *18*, 16127 https://pubs.rsc.org/en/content/articlelanding/2016/cp/c6cp00341a, accessed on 15 December 2021 (supporting information) with permission from the PCCP Owner Societies.

**Figure 5:**
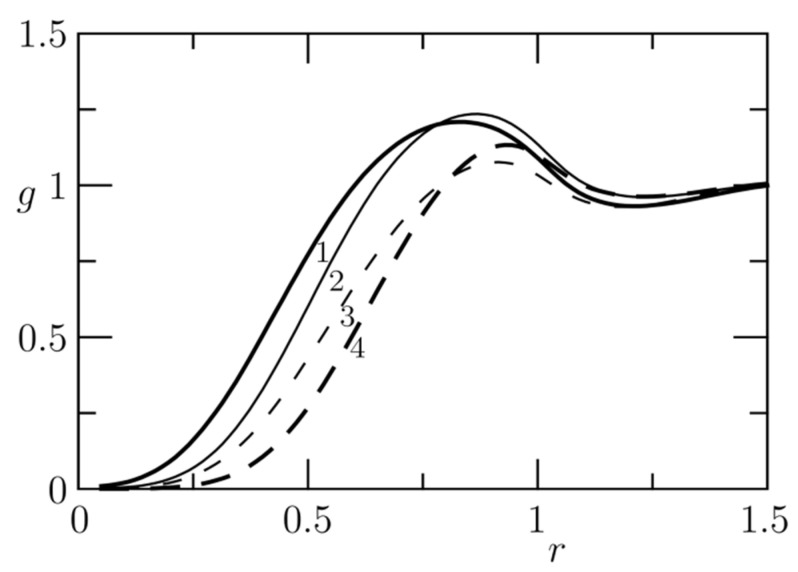
The radial distribution function, *g*(*r*), for the mixture of evenly and oppositely charged beads. The thick lines (1, 4) correspond to *λ* = 0.2, while the thin lines (2, 3) represent larger smearing with *λ* = 0.6. The full lines (1, 2) are radial distribution functions between particles bearing unlike charges, *e*_+−_, and the dashed lines (3, 4) are the radial distribution functions between particles with like charges, *e*_++_. Reproduced from *Phys. Chem. Chem. Phys.* **2016**, *18*, 16127 https://pubs.rsc.org/en/content/articlelanding/2016/cp/c6cp00341a, accessed on 15 December 2021 (supporting information) with permission from the PCCP Owner Societies.

Figure 3 compares the distributions of the electric charge in space for *λ* = 0.2 and *λ* = 0.67. In the latter case, an important part of the charge spreads outside the bead. Figure 4a,b depict the *r*-dependence of the total pair interaction potential (and of its electrostatic and non-electrostatic parts) and Figure 5 shows the effects of electrostatics on the radial distribution function of evenly and oppositely charged beads for *λ* = 0.2 and 0.67. It is obvious that the narrower charge smearing generates a stronger local effect, but the shapes of the corresponding curves are fairly similar to each other.

## 5. Recent Progress in DPD Modelling of Polymer and PE Self-and Co-Assembly

A number of interesting papers on DPD modelling of polyelectrolytes and surfactants, and particularly on their organisation, self- and co-assembly, have been published in recent decades. They focus on different research topics and can be divided roughly into several categories: (i) methodology, development and improvement of the simulation strategy and simulation machinery (improved codes, development and comparison of different interaction potentials, more appropriate parametrisation of forces, etc.); (ii) general studies of the most important trends of association processes in a wide range of conditions (experimentally inaccessible features of the behaviour, detailed study of the interplay of several contributions that cannot be separated in experimental studies, etc.); (iii) the use of DPD in combination with experimental research to support the validity of conclusions or to complete the experimentally inaccessible (or difficult of tediously accessible) pieces of knowledge and (iv) studies aimed at the development and better understanding of specific biomedical and technological applications. Because most authors combine several aspects in one study, in the next section, we do not follow the outlined categorisation but discuss the papers according to their main contribution to polymer science.

## 6. Studies Aimed at the Improvement of DPD Methodology and at Important Trends of the Behaviour

As this review focuses primarily on the electrostatic co-assembly, the papers by Gonzalez-Melchor et al. [161,165] deserve to be mentioned first. Using the potential of electrostatic interactions between smeared charges expressed by Equation (5), the authors applied the Ewald summation [166] for the enumeration of long-distance interactions and proposed a suitable alternative to the original method developed by Groot [102]. Their simulations provided data that compare well with those of Groot and the paper can be considered a seminal DPD paper.

**Figure 6:**
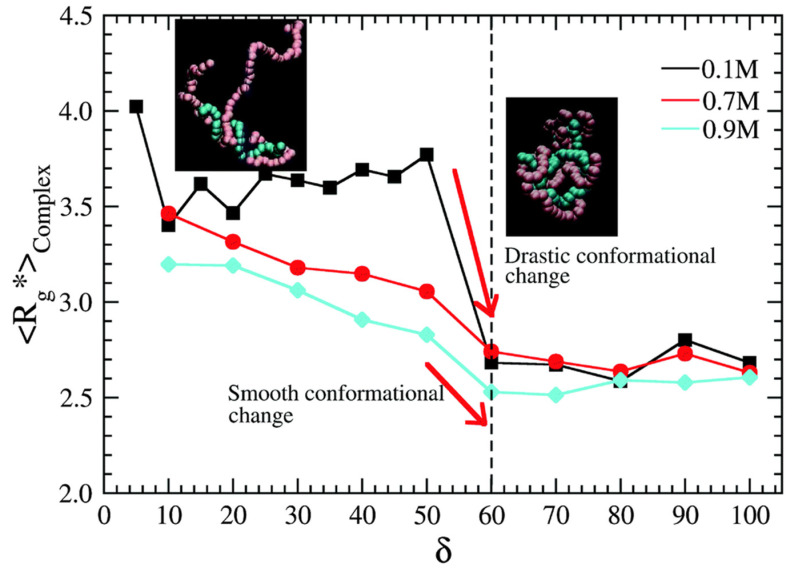
Radius of gyration of the complex as a function of PSS−size and ionic strength of the solution. The vertical dotted line indicates the ratio at which a change from an extended to a compact complex structure appears for the systems with 0.1 M of NaCl. The arrows indicate the regions of crossover from a drastic to a smooth conformational change. The insets on the left and right show the polyelectrolyte complex for δ = 30% and 60%, respectively, at 0.1 M NaCl concentration. (Republished with permission of Royal Society of Chemistry, from E. Meneses-Juarez, C. Marquez-Beltran, J.F. Rivas-Silva, U. Pal, M. Gonzalez-Melchor, The structure and interaction mechanism of a polyelectrolyte complex: a dissipative particle dynamics study. *Soft Matter* **2015**, *11*, 5889–5897, doi:10.1039/C5SM00911A. Copyright 2021; permission conveyed through Copyright Clearance Center, Inc.).

Using their method, Gonzalez-Melchor et al. later investigated the formation of electrostatically stabilised complexes between oppositely charged copolymers. They focused on the conformations of polycations and polyanions in nonstoichiometric mixtures and found that a significant chain collapse occurs at the 60% charge compensation (see Figure 6). They also studied the screening of electrostatic interaction by small ions and observed the weakening of the aggregation tendency with the increase in ion concentration, which agrees with experimental observations [167,168,169,170]. The paper provides important information and is very interesting, but it is a pity that the authors neither reported the dependences of association numbers on the composition of the mixture and on the salt concentration, nor confronted their results with the famous van der Burgh [61] speciation diagram.

The problem of the appropriate treatment of non-diverging electrostatic forces is one of the key questions of the successful DPD simulation of electrically charged systems and, therefore, several authors later tested the use of other non-diverging potentials. Gavrilov et al. [143] studied the collapse of PE chains and the microphase separation in PE blends using several potentials that diminish at short distances and formulated the following requirements for a successful DPD simulation: (i) the potential used should be identical to the Coulomb potential at distances larger than the closest distance between the neighbouring molecules in the liquid state, (ii) it should be a smooth function of distances, (iii) it should enable reasonably long integration steps and (iv) it should prevent significant overlap of oppositely charged beads.

A few years later, Gavrilov et al. [171] investigated polar systems (formally uncharged) using their DPD method with the electrostatics developed earlier. The polar units were modelled as oppositely charged dumbbells (dipoles) orientated either parallel with respect to the chain (i.e., incorporated into the chain) or perpendicular, i.e., semipendant (one part inside the chain and the other outside). The authors concluded that this approach is suitable for modelling polar polymers in polar solvents. They claim that the method ‘allows one to deal with polar species explicitly, without the need to introduce local polarisability, making it a powerful and robust tool’ for studying polar polymer systems.

Applying the mean field calculations and DPD with explicit electrostatics, Rumyantsev et al. [172] studied the phase segregation in systems of oppositely charged polyelectrolyte blends. The study shows that the driving force derives from the incompatibility of uncharged monomer units and the electrostatics strongly hinder the formation of segregated domains and control their size. A certain drawback of the study lies in the fact that the authors studied polymer systems without added counterions. Such systems can be prepared, but most polymer blends form upon mixing two polyelectrolyte salts and contain equivalent numbers of oppositely charged counterions. Nevertheless, the study captures all decisive features of electrostatic co-assembly and correctly indicates the trends of the behaviour.

An efficient DPD-based algorithm for simulating an electrolyte solution, which treats the counterions as a dynamic and fluctuating ion cloud and enables the fast evaluation of electrostatic contribution, was proposed by Medina et al. [173]. The method was elaborated for low-molar-mass electrolytes and its function was tested by calculating the electroosmotic flow in capillary electrophoresis, but the algorithm is suitable also for studying high-molar-mass systems.

A slightly problematic paper on the behaviour of polyelectrolytes at the water–oil interface was published by Nair et al. [174]. The authors set the parameters of non-electrostatic interactions to reproduce the scaling properties of neutral polymers in water and in oil and then they studied the effect of charges on the polymer and the effect of counterions. However, they did not describe the treatment of electrostatic interactions (the employed electrostatic potential, evaluation of the contribution of distant ions and more), which strongly obscures the results and conclusions.

Even more problematic are the papers published by Wang et al. [175] and by Guo et al. [176]. The authors of the first paper simulated the pH-dependent behaviour of a triblock copolymer composed of a water-soluble and a pH-sensitive (ionisable) block attached to a strongly hydrophobic block. They used simulations without explicit electrostatics and simply ascribed different parameters to the charged and uncharged units and then were able to emulate experimental data.

In the second article, the solubilisation of doxorubicin into pH-sensitive copolymer micelles composed of a cholesterol block, polyarginine and poly(histidine) block was studied by DPD simulations. Analogously to the previous paper, the increased solubility of histidine units upon the protonation was treated by a significant decrease in the repulsion parameter between the protonated histidine and water. We are of the opinion that simulations of polyelectrolytes that do not take into account real physics and ignore electrostatic interactions [176,177,178,179,180,181,182,183,184,185,186], even though they are fast and can emulate some properties (if the interaction parameters are suitably set to reproduce experimental data), fail to describe correctly the general trends of the behaviour (not speaking of their prediction), simply because they do not capture the behaviour of counterions, the entropy of which acts as a decisive driving force of the electrostatic co-assembly.

**Figure 7:**
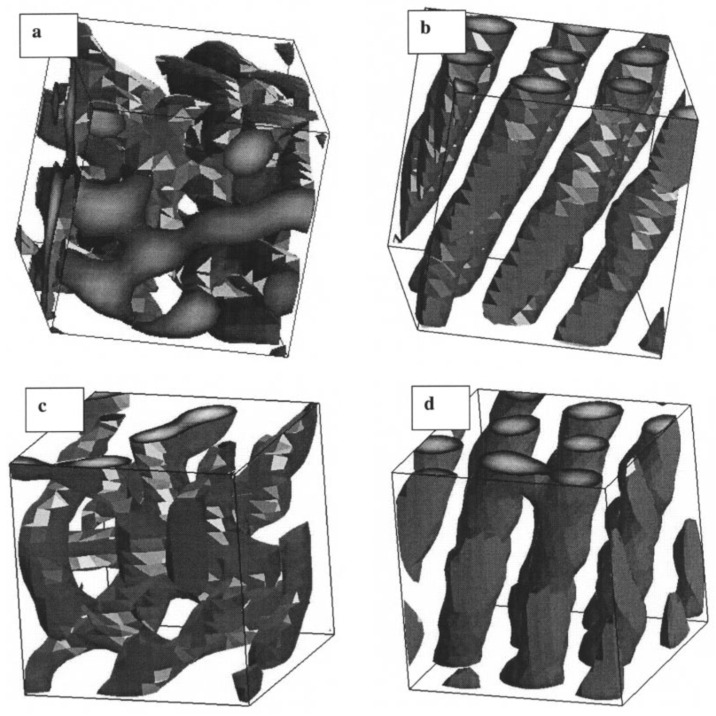
Morphological evolution of hexagonal cylinder phase obtained using DPD and soft BD simulations with *N* = 27,000, *f*_A_ = 0.3 and *r* = 3.0 *r*_c_^−3^. (**a**) Interconnected tube structure obtained using DPD with *t* = 1000 τ and (χ*N*)_eff_ = 23.7. (**b**) Hexagonal cylinder phase obtained using DPD with *t* = 30 000 τ and (χ*N*)_eff_ = 23.7. (**c**) Interconnected tube structure obtained using soft BD with *t* = 1000 τ and (χ*N*)_eff_ = 23.7. (**d**) Hexagonal cylinder phase obtained using soft BD with *t* = 150,000 τ and (χ*N*)_eff_ = 23.7. (Copyright *J. Chem. Phys.*, doi:10.1063/1.1814976).

Concerning the DPD methodology, an important article was published by Horsch et al. [187]. The authors addressed the phase behaviour of melts of block copolymers with the finite length. They used several computer-aided methods and compared the predictions from the FH theory with numerical results of molecular dynamics, Brownian dynamics with and without hydrodynamics and with results of DPD and proposed a relationship between the parameters of the Lennard-Jones potential and DPD parameters. They focused on the formation of the hexagonal cylinder phase (see Figure 7) and, in contrast to an earlier study [188], found that its formation does not require the action of hydrodynamic forces, as was believed earlier.

Important stimuli for the optimisation of coarse-grained methods come from the multiscale studies and from the papers that compare the results of DPD with other types of simulations and/or with mean-field predictions [189,190,191,192,193,194,195]. Spaeth et al. [196] employed the implicit solvent Brownian dynamics (BD) using the parameters proposed earlier by Chen [197] and the explicit solvent DPD simulation. The authors emulated the experiment by Kumar [198], who prepared stable core–shell polystyrene–*b*-poly(ethylene oxide) PS–*b*-PEO nanoparticles by fast nanoprecipitation. The study showed that both methods reproduced the experiment at the semiquantitative level and yielded similar aggregates (even though they were smaller than those formed in experiments); only the BD-generated PEO blocks were more expanded and the formation of associates and their growth proceeded more slowly in BD than in DPD.

Some papers comparing different methods were devoted to the dynamics of PE chains under flow. Even though we are interested in static properties, we feel obliged to mention the paper by Jayasree et al. [199]. The authors compared the PE conformations under flow generated by Brownian dynamics with full electrostatics and results of DPD simulations with only the long-range part of electrostatic interactions treated by the Ewald summation. They found reasonable agreement, which they attributed to the fact that the fast and short-range dynamics of PE chains are strongly coupled with those of counterions. The paper thus provides information that is important for DPD methodology and for the numerical solution of equations of motion of charged species.

The flexibility of polymer and surfactant chains varies significantly and affects the structure and behaviour of assembled nanodomains. Therefore, it is not surprising that this topic attracted the interest of many research groups. The semiflexible chains are usually modelled using the angle-dependent potentials, restricting the range of mutual orientations of two (or more) successive bonds [127,128]. An alternative approach was applied by the Neimark group, who addressed the effect of the restricted flexibility of surfactants on their self-assembly in several publications [121,135,200]. They did not apply the angular potentials, but, in addition to the bond forces between beads *i* and *i* + 1, which they modelled either by harmonic or by FENE potentials, they added analogous potentials between beads *i* and *i* + 2 and *i* and *i* +3, confining the average distances between them to relatively large values and thus stretching the chain. Quite recently, the group used another interesting approach to the treatment of the angularly dependent coordination of multivalent metals to polyelectrolyte chains using the predefined directions in the coordination sphere of the metal atom [201].

## 7. Experiment-Inspired and Application-Orientated Papers

Numerous papers were inspired by experimental studies and aimed at prosperous applications of self-assembling polymer and polyelectrolyte systems in various fields. They offer the explanation of experimental results at the molecular level and provide details inaccessible by experiments, including various specific application-orientated pieces of information.

Great attention has been devoted to assembly in copolymer solutions and melts, to the structure of assembled nanoparticles and to morphological transformations upon variation of solvent composition, polymer concentration, ionic strength, etc. The behaviour of flexible polyelectrolytes and/or amphiphilic copolymers [69,91,105,106,174,178,193,202,203,204,205,206], copolymers composed of linear and rigid blocks [127,128,207,208] and also hyperbranched copolymers [209,210,211] has been simulated and the decisive principles and trends of underlying processes have been described. Besides the above systems, the multicore assemblies forming in mixtures of linear copolymers [212], the self-assembling amphiphilic homopolymers [213], side-chain discotic liquid crystalline polymers [214], polymers tethered to nanoparticles and mixtures of copolymers with nanoparticles [215,216] and the ‘shape amphiphiles’ [217,218,219,220,221] have been successfully studied by DPD. Because the most promising and very intensively studied application of polymeric nanoparticles is the targeted transport of drugs in living organisms and their controlled release [17,21,23,25], several DPD and combined experimental–DPD studies have also been published recently [176,177,183,222,223,224,225]. Concerning biomedical applications, the relatively recent reviews by Biswas [225] and Chen [226], and particularly the references therein, are of interest. However, it is a pity that a high fraction of biomedically orientated DPD studies of pH-dependent ionised systems do not use the explicit electrostatics, and this drawback significantly weakens their importance.

**Figure 8:**
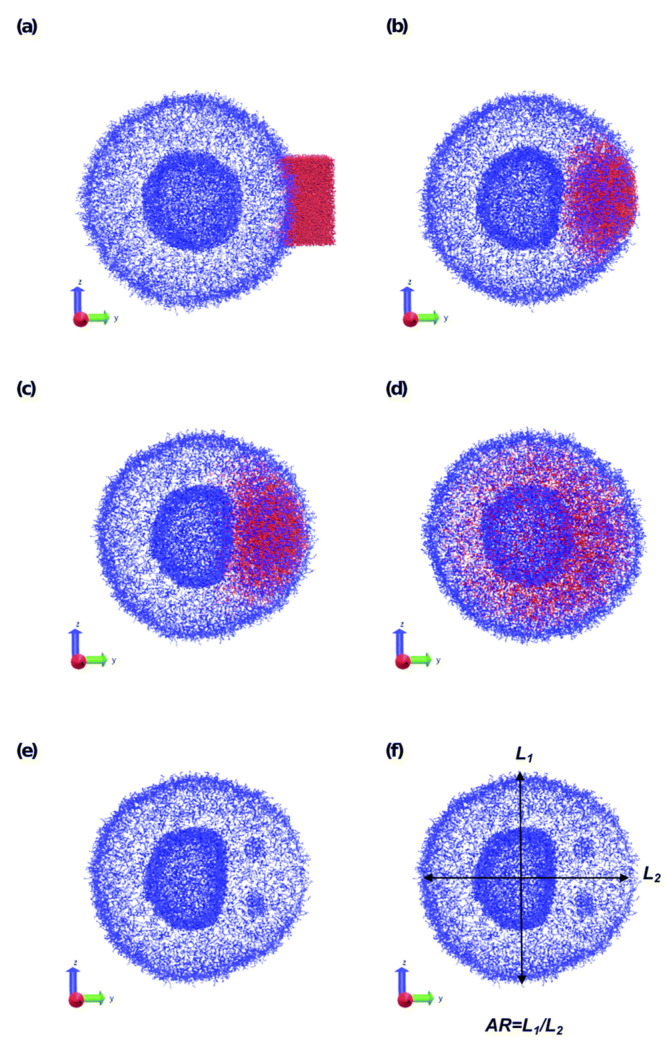
Microinjection of a droplet of SB that is 8.07% of the vesicle volume (15,584 beads) near the surface of the vesicle at (**a**) *t* = 0, (**b**) *t* = 200, (**c**) *t* = 500 and (**d**) *t* = 5000. The polymer A block is shown in blue, SB is coloured red and the polymer B block and SA beads are not shown. Panel (**e**) shows *t* = 500 again, omitting SB to highlight the flattening of the lumen wall and the formation of a micelle in the inner membrane. Panel (**f**) defines the aspect ratio (AR) as the ratio between the spans in the *z*-direction and the *y*-direction. (Republished with permission of Royal Society of Chemistry, from Q.Y. Zhu, T.R. Scott, D.R. Tree, Using reactive dissipative particle dynamics to understand local shape manipulation of polymer vesicles. *Soft Matter* **2021**, *17*, doi:10.1039/d0sm01654c; Copyright 2021; permission conveyed through Copyright Clearance Center, Inc.).

**Figure 9:**
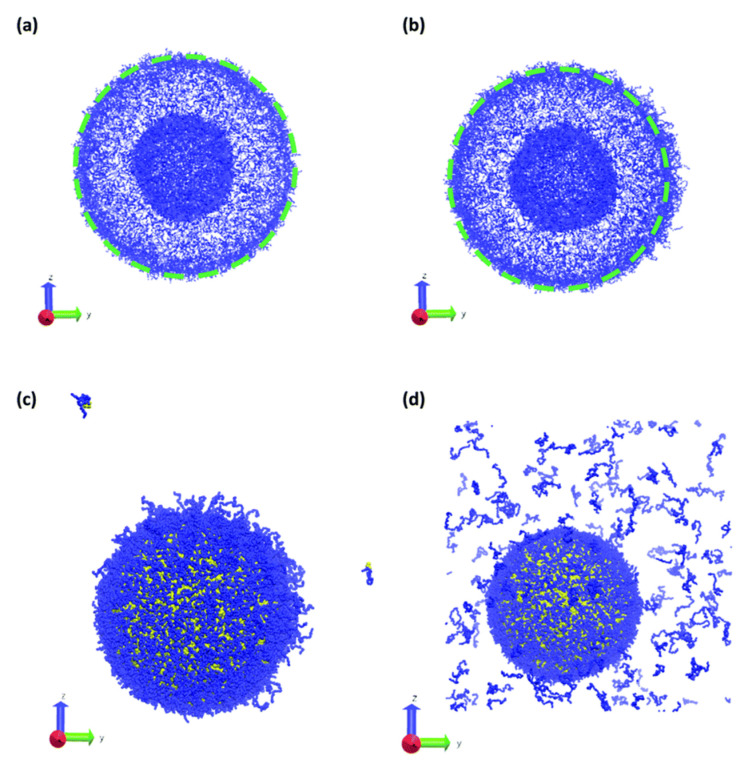
Vesicle morphology at *t* = 2.5, 104 following an instantaneous change at *t* = 0 of the block fraction of a localised portion on the right side (positive *y*-direction) of the vesicle from *f*_A_ = 0.2 to (**a**) *f*_A_ = 0.1, (**b**) *f*_A_ = 0.4, (**c**) *f*_A_ = 0.8 and (**d**) *f*_A_ = 1.0. As above, blue beads show A-type monomers, and, in panels (**c**,**d**), yellow beads represent B-type monomers. Solvent beads are not shown. The green circle in panels (**a**,**b**) shows the circumference of the original vesicle as a guide to the eye. (Republished with permission of Royal Society of Chemistry, from Q.Y. Zhu, T.R. Scott, D.R. Tree, Using reactive dissipative particle dynamics to understand local shape manipulation of polymer vesicles. *Soft Matter* **2021**, *17*, doi:10.1039/d0sm01654c; Copyright 2021; permission conveyed through Copyright Clearance Center, Inc.).

The number of papers on the soluble self-assembled polymeric particles is very high and we will discuss only a few of them in more detail, especially those addressing charged systems and exploiting explicit electrostatics. An interesting paper was recently published by Zhu et al. [227]. The authors combined DPD with Split Reactive Brownian Dynamics (SRBD) [228], creating the Reactive DPD (RDPD), which enables the study of reacting systems. Inspired by the behaviour of cell membranes and by papers by Wright [229], who experimentally studied the molecular rearrangements in stimuli-responsive systems and offered their computer-based interpretations, and by the simulation paper by Gumus [230] on the morphology changes of kinetically trapped polymeric nanostructures upon the addition of a nonsolvent, Zhu et al. successfully simulated the local shape changes of polymer vesicles upon the microinjection of a droplet of a good solvent, which triggers polymer swelling (see Figure 8 and Figure 9).

Posel et al. addressed the behaviour of poly(2-vinylpyridine)-*b*-poly(ethylene oxide) (P2VP-*b*-PEO) in aqueous solutions [134]. The study was motivated by a very interesting pH-dependent self-assembly and by the fact that some of the co-authors studied this self-assembly experimentally [82]. PEO is a water-soluble polymer that dissolves in aqueous buffers in a wide pH range. The deprotonated P2VP is strongly hydrophobic and insoluble in neutral and alkaline aqueous solutions, but, after the protonation of the nitrogen atoms at pH < 4.8, the charged P2VPH^+^ becomes readily water-soluble. The copolymer forms spherical core–shell micelles with hydrophobic P2VP cores in alkaline and neutral solutions. In acidic solutions, the micelles readily dissociate and the copolymer molecularly dissolves because both PEO and the protonated P2VPH^+^ are highly soluble below pH 4.8. The formation/dissociation of micelles upon a slight pH change are reversible and reproducible processes. As the sharp change in P2VP solubility in a narrow pH range occurs without an appreciable chemical change in the polymer building units (it is a result of the proton dissociation/association only), the system is ideal for the testing and proper setting of the balance of non-electrostatic and electrostatic forces. Experimental molar masses, *M*_W_ (or association numbers), of micelles formed in alkaline solutions enable the adjustment of the parameters of non-electrostatic interactions of P2VP and the comparison of the pH-dependent dissociation of micelles emulated by DPD (using the experimental acidic dissociation constant, *K*_A_^ap^, of P2VP) with the corresponding experimental curve provides a feedback control of the proper setting of the balance between non-electrostatic and electrostatic forces.

**Figure 10:**
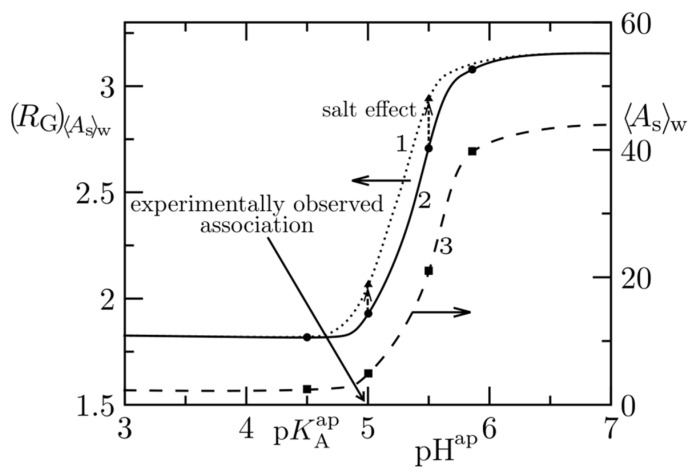
Weight average association number 〈*As*〉_W_ (dotted curve 3) and apparent radius of gyration, (*R*_G_)_〈__*As*__〉__w_, (solid curve 2) as functions of pH^ap^. The effect of the salt is depicted by the shift in the radius of gyration for ionic strength *I* = 0.1and *I* = 0.25 (dotted curve 1). (Reprinted with permission from *Macromolecules* **2014**, *47*, 2503–2514, doi:10.1021/ma402293c. Copyright 2021 American Chemical Society).

The most important results of the simulation study are reproduced in Figure 10.

In summary, the simulation study of the P2VP-*b*-PEO self-assembly, based on the simplifying assumption that the average ionisation of P2VP units is proportional to the pH-dependent ionisation degree, *α*, which can be enumerated from the definition of the dissociation constant, *K*_A_^ap^ (of protonated beads behaving as independent univalent acids),

(6) $$\mathrm{pH}=pK_{A}^{\mathrm{ap}}+log\frac{\alpha }{1-\alpha }$$

confirmed that the description used of electrostatic interactions with the charge decay constant, *λ* = 0.2, and the parameters *a_ij_* = 40 of non-electrostatic repulsive interactions (i) between the non-protonated hydrophobic P2VP units and water and (ii) between mutually incompatible hydrophobic P2VP and hydrophilic PEO blocks nicely reproduce the experimentally observed association behaviour.

The other example of the fine tuning of electrostatic vs. non-electrostatic interaction parameters [231] concerns the study of the solubilisation of ionic porphyrin derivatives (PR) in the interpolyelectrolyte complex (IPEC) cores of block PE micelles. The PR solubilisation in nanoparticles finds practical applications in medicine (in photodynamic therapy), but, surprisingly, the embedding of ionic porphyrin derivatives in IPEC complexes almost escaped the interest of researchers. PR are little soluble in aqueous media and form several types of aggregates depending on the concentration, ionic strength, pH, etc. The aggregation is a result of important *π-π* stacking. The attachment of pendant ionic groups improves the solubility and restricts the aggregation tendency. The multiple charged PR (bearing 2 to 4 charged groups) are usually reasonably soluble in low-ionic-strength buffers, where they predominantly dissolve as monomers. Increasing the salt concentration screens the electrostatic interaction, deteriorates the solubility and promotes the aggregation. The ionic-strength-dependent experimental data on the solution behaviour of ionic PR in aqueous media were used as the calibration gauge for the appropriate setting of interaction parameters. Using the electrostatic potential described by Equation (5) and exploiting the strategy of mapping DPD results onto the concentration-dependent and ionic-strength-dependent experimental data on porphyrin solutions [232,233,234], the authors obtained the optimum parameters for non-electrostatic porphyrin–porphyrin, porphyrin–water, porphyrin–pendant group and pendant group–pendant group repulsion parameters, which they further used for predicting PR solubilisation in IPEC complexes.

**Figure 11:**
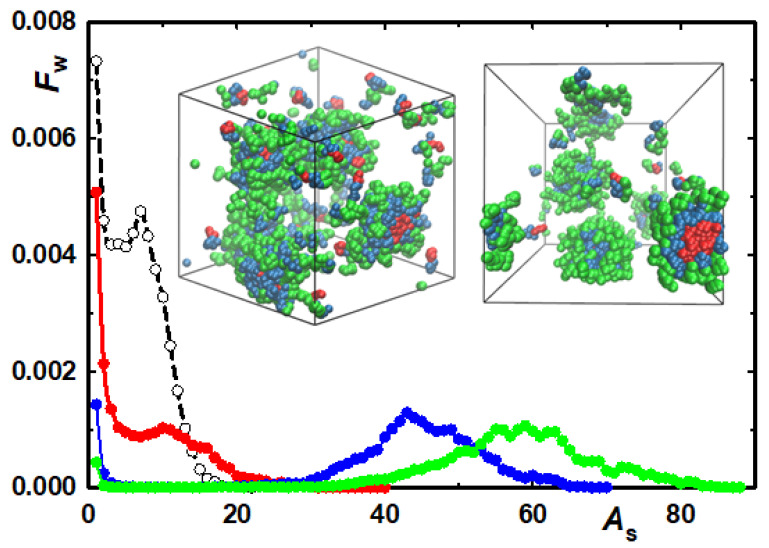
Weight distribution function, *F*_w_(*A*_s_), of association numbers, *A*_s_, for the pre-aggregated micelles A_5_B_5_^+^ with Δ*a*_BS_ = 0 (B block compatible with the solvent—black dashed curve), for onion micelles formed by A_5_B_5_^+^ micelles and C_7_B_5_^−^ chains with Δ*a*_BS_ = Δ*a*_BC_ = Δ*a*_BI_ = 0 (B block compatible with the solvent, with block C and with counterions—red curve) and for systems with increasing compatibility of blocks (Δ*a*_BS_ = Δ*a*_BC_ = Δ*a*_BI_ = 3.5—blue curve and Δ*a*_BS_ = Δ*a*_BC_ = Δ*a*_BI_ = 5.0—green curve). The left-hand-side insert shows a typical snapshot of associates with Δ*a*_BS_ = Δ*a*_BC_ = Δ*a*_BI_ = 3.5 and the right-hand-side one shows a snapshot for the value of 5 (beads A—red, beads B—blue and beads C—green). (Reprinted with permission from *Macromolecules* **2020**, *53*, 6780–6795, doi:10.1021/acs.macromol.0c00560, Copyright 2021 American Chemical Society).

An interesting DPD study of the complex process comprising the combination of electrostatic co-assembly and amphiphilic self-assembly was recently published by a team of experimentalists and computer scientists. They studied the formation and properties of onion micelles prepared by mixing preformed core–shell micelles composed of hydrophobic cores and cationic polyelectrolyte shells with a double-hydrophilic block copolymer composed of an anionic block and a water-soluble poly(ethylene oxide) block [235]. The simulation proved that the three-layer micelles with the compact central hydrophobic block, compact middle interpolyelectrolyte complex layer and the diffuse stabilizing water-soluble shell form not only in mixtures of pre-aggregated core–shell micelles, but also upon the transfer of the components dissolved in a common solvent into a selective aqueous medium. An example of the simulation results is reproduced in Figure 11.

Steric confinement strongly influences the assembly of polymers and represents a powerful tool for controlling and manipulating the shape and internal structure of created nanoparticles. The strategy of preparation of novel nanostructures under confinement was exploited by a number of experimental research groups [236,237,238,239,240]. More examples can be found in the review by Yabu [241]. The impact of the confinement and the trends of the behaviour were also studied theoretically by SCF [242,243,244] and by computer simulations, including DPD [245,246,247].

A non-negligible fraction of DPD papers were devoted to studies of various block copolymer membranes (semipermeable, porous and others), narrow films and tethered polymer brushes. From a theoretical point of view, the flat and low-curvature surfaces and interfaces represent a mild 1D confinement sterically restricting polymer conformations. Here, we discuss the porous membranes in more detail because they find important applications in many biomedical and technological areas. A fairly reproducible, controllable and simultaneously relatively facile method of preparation of porous membranes consists of the dissolution of a suitable copolymer (usually a hydrophobic–hydrophilic one) in a common solvent for both blocks and in a fast transition into a solvent (commonly water) that is poor for the hydrophobic block. In solutions containing ca. 30–40% of the copolymer, the self-assembly process leads to fairly regular membranes consisting of a dense hydrophobic matrix perforated by regularly arranged spherical holes with a surface covered by the soluble blocks. This method, called non-solvent-induced phase separation (NIPS), was successfully applied by a couple of experimental groups [248,249,250,251]. Several authors used DPD to confirm the self-assembling mechanism of NIPS proposed by experimentalists, focusing on the effects caused by the concentration, lengths of blocks and others on the structure, regularity and stability of the prepared membranes [252,253,254,255].

Recently, Karunakaran [256] succeeded in preparing porous membranes also in systems of double-hydrophobic copolymers. In contrast to the previous system, the mechanism of membrane formation is not clear. Jiang et al. [257] performed an extensive study of a series of polystyrene-*b*-poly(methyl methacrylate), PS_5_-*b*-PMMA_25_ to P30_5_-*b*-PMMA_25_ samples in good and bad solvents. Using a GALAMOST DPD method [258] in a large 100^3^ box (recalculated according to a standard volume of water to a 65^3^ box), they studied the effect of concentration, length of blocks and the quality of the common solvent. They found that fairly regular porous membranes form spontaneously in solutions containing 30–40% of the polymer component. In dilute solutions, small spherical aggregates with PS cores form, and, in highly concentrated solutions, various irregular dense structures form. The study indicates that the polymer concentration is the crucial factor for the successful preparation of porous membranes. The relative length of blocks and the choice of the common solvent (interacting differently with individual blocks) affect the structure of the membrane (e.g., size of the pores), but both factors play only a minor role because stable membranes form in all studied systems in the region between 30% and 40% of polymer content. The study reveals that the success of the method comes from the difference in hydrophobicity between both blocks and elucidates both the slightly unexpected mechanism of the self-assembly and the good reproducibility of the preparation porous membranes.

A few years ago, the area of functional zwitterionic membranes was addressed by the research group of Zhou. They studied the pH-responsive zwitterionic membranes formed by hydrophobic–hydrophilic diblock copolymers decorated by a short, rigid, hydrophobic sticker [259]. The DPD study with explicit electrostatics is interesting and shows the general self-assembling behaviour of this type of copolymer. However, the conclusion that the authors designed and developed a new system based on docosahexaoinic acid-*b*-poly(*γ*-benzyl L- glutamate-*b*-poly(carboxybetaine methacrylate) suitable for drug (doxorubicin) delivery and investigated its properties is, in our opinion, unjustified. This claim is not supported by experimental data and the authors used only the coarse-grained DPD, applying the repulsion parameters for the nonpolar interactions from Material Studio 7.0, thus ignoring all potential chemistry caprices.

A recent study of the same research groups addresses membranes for separation technologies. Membranes based on polyvinylidene fluoride (PVDF) decorated by zwitterionic polymer brushes are suitable materials, e.g., for seawater desalination, wastewater treatment and more. As it was reported that adding silica particles during their preparation improves their mechanical stability [260] and that the decoration of inorganic particles by zwitterionic polymer brushes enhances the compatibility of the components [261,262], the group performed a study of the formation of mixed membranes [263]. They found that grafted nanoparticles gradually migrate to the surface of the membrane and form a fairly uniform layer there.

The formation, structure and properties of nanostructured membranes are also very important topics for modern lithography. Stoychovich et al. have shown experimentally that the directed assembly of copolymers on chemically patterned surfaces can produce useful structures for the fabrication of integrated circuits [264]. Inspired by this study, Takahashi et al. [265] performed targeted DPD simulations, reproducing the copolymer self-assembly in trench and cylindrical holes. They optimised the level of coarse-graining, the bond parameter and the interaction parameters and obtained reasonable agreement with experimental data. Even though the parametrisation was based on physical principles, the authors focused strictly on one experimental system only and, being aware of this weak point, they neither discussed general trends nor offered any predictions of the behaviour of other membranes used in lithographic procedures.

Other types of important polymeric materials are the hydrated selectively permeable membranes (particularly the Nafion membranes), which play a crucial role in fuel cells. Their study has represented a hot topic for experimentalists [266,267], theoreticians and computer scientists for several decades, and it is not surprising that several recent DPD studies have addressed the fuel cell membranes [124,253,268,269,270,271,272,273]; the last two papers unfortunately study the electrically charged system without explicit electrostatics, which restricts their impact. On the other hand, the papers on hydrated membranes published by the Neimark group [124,271,273] are of special interest because they also contribute significantly to the methodological progress in DPD modelling. Using the short-range Morse potential for the interaction of protons with water molecules, the authors developed a suitable model for proton dissociation, which extends the use of DPD for reactive systems.

Relatively recently, DPD was applied to emulate and elucidate the chemoepitaxy and graphoepitaxy preparation of thin films [274]. The authors reparametrised the density, bond distance, inter-bead potential and noise ratio. After they increased the system density to 5 and set *r*_0_ = 1 and *K* = 40 in the harmonic bond potential, they were able to reproduce the structures formed in real systems by their modified DPD simulations.

**Figure 12:**
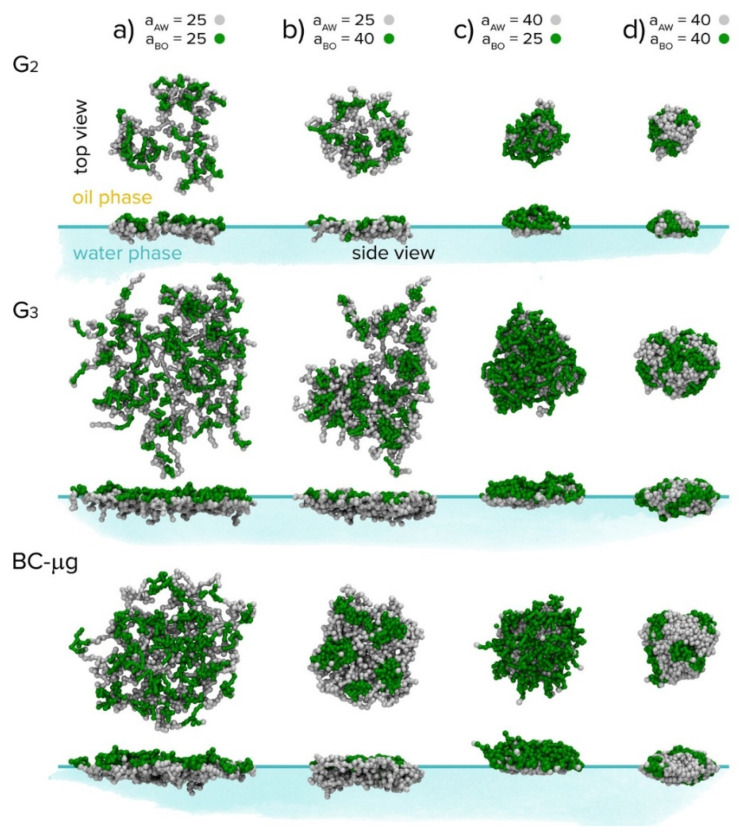
Snapshots of single arborescent copolymers and block copolymer microgel adsorbed at the interface of two immiscible liquids. (**a**) First column corresponds to the case when the upper (oil) and bottom (water) liquids act as good solvents for the green (B) and the grey (A) beads, respectively, and vice versa (*a*_AW_ = *a*_BO_ = 25, *a*_AO_ = *a*_BW_ = 40). (**b**) Second column shows the regime when water is a good solvent for the A beads, poor solvent for the B beads and oil is a poor solvent for both polymeric beads (*a*_AW_ = 25, *a*_BO_ = 40). (**c**) The third column represents the swelling of minor (B) component in the oil (*a*_BO_ = 25, *a*_AW_ = 40). Finally, (**d**) the fourth column demonstrates deformation of the molecules insoluble in both liquids (*a*_AW_ = *a*_BO_ = 40). (Reprinted with permission ACS from *Appl. Mater. Interfaces* **2017**, *9*, 31302−31316, doi:10.1021/acsami.7b00772. Copyright 2021 American Chemical Society).

The behaviour of copolymers and polymeric nanoparticles at interfaces is not confined only to membranes and thin films. It covers a broad spectrum of phenomena important from the viewpoint of colloid, polymer and interface science and offers various technological applications. Consequently, a number of theoretical computer studies included multiscale approaches and DPD (see the Special Issue of *Interface Science* published in 2020 [275,276]). Guskova et al. [277] studied the directed assembly of polymer-grafted nanoparticles on the surfaces of phase-separated copolymer brushes and reported the formation of various regularly arranged nanodot and nanowire systems. The authors offered some rules facilitating the design and optimisation of this class of nanomaterials. Another interesting paper was published by Gumerov et al. [278]. The authors studied the behaviour of arborescent (dendrograph) copolymers at the oil–water interface. The arborescent copolymers are highly branched high-molar-mass dendritic molecules, first prepared and studied by cooperating Gautier and Möller teams [279] and independently by Tomalia et al. [280]. They contain linear sequences of monomer units differing in solvophilicity/solvophobicity and offer a number of potential applications as templates for the preparation of metallic nanoparticles [281], drug delivery [282] or as efficient additives for polymer processing [283]. In agreement with experimental observations, the study shows that the arborescent copolymers of the second and third generation are fairly spherical in common, good solvents. In selective solvents, they almost do not aggregate, but form unimolecular micelles. They flatten at the liquid–liquid interfaces and their conformation and properties depend on the quality of the solvents used. Depending on their amount in the system, they form both loose and dense nanostructured or homogeneous films at interfaces and the study indicates that they can be used as efficient emulsifiers. The conformations created at interfaces are depicted in Figure 12. Qualitatively similar behaviour of flexible polymeric dendrimers at interfaces in porous media was recently reported also by X. Wang et al. [284].

The simulation trajectory starting from a random mixture of molecularly dispersed chains emulates the time evolution of the self-assembling system at the DPD level. If the coarse-grained model captures all decisive features of the real system and all parameters have been well set, the time evolution of the simulated system provides valuable information on the growth of aggregates and on the parameters that affect it. Hence, several authors have studied the kinetics of the aggregation process and investigated the role of the polymer chain architecture, relative lengths of blocks, solvent quality and others [229,285,286,287].

Recently, Ye at al. [288] published the results of large-scale DPD simulations in simulation boxes 100^3^ and 150^3^ containing 20k to 80k copolymer chains (A_12_B_6_ to A_12_B_8_). They used a LAMMPS code and performed massively parallel simulations. They studied the time evolution of the self-assemblies and observed the step-wise growth of nanoparticles formed by the fusion of smaller ones. First, relatively small spherical and rod-like micelles formed in the selective solvent; later, they transformed into membrane-like assemblies, which finally bent and formed the vesicles. The final stages usually contained mixtures of various types of nanoparticles, including single-wall and multilayer vesicles and core–shell and onion micelles. The results are interesting. The simulations revealed the presence of low fluctuating fractions of single chains and the authors claim that it is the first time that the simulations have shown the dynamic exchange of chains. This statement is incorrect because the dynamic exchange of unimer chains among micelles was reported earlier, e.g., in papers based on the cooperation of the Procházka and Lísal groups [67,69,105,106,134,289]. Moreover, Ye et al. used fairly high repulsion parameters (*a*_AB_ = *a*_AS_ = 50) to model the incompatibility of the A and B beads and the insolubility of the solvophobic beads A vs. the standard value *a_ii_* = 25 for the similar beads. Therefore, it is unclear whether the ultimate simulation stage in systems with longer insoluble blocks than soluble ones could be the macroscopic precipitation of the copolymer and how the shape, size and association number of associates would change in systems with lower repulsion parameters. Some simulations of other authors (e.g., the Lísal and Procházka group) suggest that the worsening of the thermodynamic quality of the solvent and the consequent retardation of the dynamic exchange of chains between associates can lead to a stepwise increase in the association numbers simply because the system remains arrested for relatively long times in fairly frozen states. Nevertheless, information on the fusion mechanism is interesting and the paper is worth studying.

## 8. Summary and Concluding Remarks

The main purpose of this critical review is to inform the broad community of polymer scientists on the coarse-grained DPD computer simulation method, with its impact on polymer science and particularly on its contribution to the field of self-organisation and self-assembly of amphiphilic copolymers and polyelectrolytes. In the first sections, we elucidate the principles of DPD simulations from the viewpoint of general polymer physics, trying to explain the essential assumptions and some mathematical tricks to lay people in computer science. In contrast to the recent well-written, methodology-orientated reviews [84,290], we focus on the results of recent papers and on their contribution to the broadening of knowledge on self- and co-assembling processes in systems containing polymers, polyelectrolytes and surfactants. In the second half of the paper, we review DPD papers published in roughly the last two decades, analysing their usefulness for polymer science and for biomedical and various technological applications.

The enormous number of successful DPD studies on polymer systems and on processes of their spontaneous assembly and organisation show that DPD is robust and simultaneously versatile for studies of polymer systems. Not only has the power of state-of-the-art computers increased tremendously in recent years, but the methodology of DPD simulations has also improved considerably and various studies of large complex systems are now possible. There are prescriptions and databases for the relatively accurate setting of interaction parameters for individual biomedically or technologically interesting systems; various program packages enable simulations with different masses and sizes of DPD beads, simulations of entanglements in systems of self-avoiding chains, studies of polymers at both permeable and impermeable interfaces and sophisticated studies of electrically charged polyelectrolytes, including weak polyelectrolytes and other reversibly dissociating/associating systems. In spite of this, still, a non-negligible fraction of papers of the pH-dependent behaviour of self-assembling polymer systems published in recognised journals (particularly in biomedical ones) do not use the DPD variant with explicit electrostatics, but rather the ‘ion-free’ approach. We would like to repeat once more that the simulation model should be based on real physics and should involve all important interactions and particles contained in the system to be modelled, particularly in the case when the entropy of counterions plays a very important role and is in fact the decisive driving force of the electrostatic co-assembly.

## Data Availability

Not applicable.
